# Education and employment in patients with juvenile idiopathic arthritis – a standardized comparison to the German general population

**DOI:** 10.1186/s12969-017-0172-2

**Published:** 2017-05-22

**Authors:** Jenny Schlichtiger, Johannes-Peter Haas, Swaantje Barth, Betty Bisdorff, Lisa Hager, Hartmut Michels, Boris Hügle, Katja Radon

**Affiliations:** 1 Unit of Occupational and Environmental Epidemiology and NetTeaching, Institute and Outpatient Clinic for Occupational, Social and Environmental Medicine, University Hospital of Munich (LMU), Ziemssenstrasse 1, D-80336 Munich, Germany; 2 German Center for Pediatric and Adolescent Rheumatology, Garmisch-Partenkirchen, Germany

**Keywords:** Juvenile idiopathic arthritis, Jia, Education, Chronic disease, Direct standardization, German general population

## Abstract

**Background:**

Although several studies show that JIA-patients have significantly lower employment rates than the general population, the research on educational and occupational attainments in patients with juvenile idiopathic arthritis (JIA) remain conflicting most likely due to small sample sizes. Therefore, aim of this study is to compare the educational achievements and employment status of 3698 JIA-patients with the German general population (GGP).

**Methods:**

“SEPIA” was a large cross-sectional study on the current status of a historic cohort of JIA-patients treated in a single center between 1952 and 2010. For the analyses of education and employment a sub-cohort was extracted, including only adult cases with a confirmed diagnosis of JIA (*N* = 2696). Participants were asked to fill out a standardized written questionnaire on education and employment. Outcome measures (education/unemployment) were directly standardized to the GGP using data obtained from the National Educational Panel Study 2013 (*N* = 11,728) and the German Unemployment Statistics 2012 of the Federal Statistical Office (*N* = 42,791,000).

**Results:**

After age- and sex-standardization, 3% (95% Confidence Interval 1.9 to 4.1%) more of the JIA-patients (26%) than of the GGP (23%) had only reached primary education. In contrast, parents of JIA-patients had similar levels of education as parents in the GGP. With a standardized difference of 0.2% (95% CI: 0.16 to 0.19%), the unemployment rate in JIA-patients was slightly, but not significantly higher than in the GGP. Stratifying for disease duration and the current treatment status, differences were confirmed for persons diagnosed before 2001, whilst for patients diagnosed after 2000, differences were found only in JIA-patients with ongoing disease. Medium and high educational achievements did not differ statistically significant between JIA patients and the GPP.

**Conclusion:**

Educational achievements in German JIA-patients are significantly lower than in the GGP. Furthermore we were able to identify a slightly higher level of unemployment, especially in those with still under treatment and longer disease duration. Better treatment options as well as further development of social support programs might help to overcome this lifelong secondary effect of JIA.

**Electronic supplementary material:**

The online version of this article (doi:10.1186/s12969-017-0172-2) contains supplementary material, which is available to authorized users.

## Background

Juvenile idiopathic arthritis (JIA) is a heterogeneous multifactorial autoimmune disease [1–3], characterized by persistent joint inflammation causing severe swellings, pain and limitation of movement [3]. The current definition of JIA unifies the previous classifications juvenile chronic arthritis (JCA) and juvenile rheumatic arthritis (JRA) [2, 4]. Incidence of JIA in Germany is estimated with 35 per 100,000/year, whereas in the Unites States the incidence rate is lower with 12 per 100,000/year [5]. Despite these comparatively low incidence rates, JIA is the most common rheumatic disease in children and adolescents and one of the most important causes of disability in this age group [1, 6, 7].

Although JIA-patients are frequently affected by severe functional impairment caused by inflammation and inflammation-related complications, some studies suggest that JIA-patients have a higher school graduation and postgraduate degrees compared to the general population [7–9]. Nevertheless, research on the educational attainments in patients with JIA remains conflicting [4, 10–12]. Concerning the employment status several studies have shown that adult JIA-patients have significantly lower employment rates than their healthy counterparts [1, 7, 8, 13–15]. Other research were able to show an association between arthritis and increased employment rates [15–17]. However, for the general description of the transition process into adulthood, the results of several studies suggest, that there is no significant difference between patients with rheumatic disease and their healthy peers [18].

Inconsistent findings across studies might mainly be caused by a variable age range of the participants or limited power due to the mostly low number of JIA-patients included [7]. Therefore the different results of recent studies make further research indispensable.

Considering previous contradictions the presented cross-sectional study compares the age- and sex-standardized educational achievements and the employment status of a large sample, including 3698 JIA-patients, to the German general population (GGP).

Since, in Germany parental level of education is still a major predictor of one’s educational achievement [19], the age- and sex-standardized parental level of education of the patients and the German population was also compared.

## Methods

### Study population and study design

#### JIA patients

The SEPIA study (**S**tudie zu malignen **E**rkrankungen bei **P**atienten mit juveniler **i**diopathischer **A**rthritis) is a single-center hospital-based study. 10,580 patients who were hospitalized in the German Center for Pediatric and Adolescent Rheumatology (GCPAR) in Garmisch-Partenkirchen (GCPAR) between 1952 and 2010 were contacted. The GCPAR is the largest clinic for pediatric rheumatology in Europe with 60 years of experience it treats approximately 2500 inpatients and 900 outpatient patients a year. The clinic is focused on the treatment of children with inflammatory rheumatic diseases and chronic pain.

For our study patients were eligible if they had been diagnosed with JIA, JCA or JRA. Furthermore, they had to live in Germany and to be fluent in German. All participants had to sign an informed consent form; if they were under the age of 18 a parent/legal guardian had to give his/her permission for their participation. The Medical Ethical Committee of the University Hospital of Munich (LMU) approved the study. (Project- Nr.: 226-11).

#### General population

Data on the education of the German general population was extracted from the National Educational Panel Study (NEPS). In addition, the German Employment Statistics 2012 including the German population aged between 20 and 64 years were obtained from the Federal Statistical Office.

### Recruitment

After a pilot test for comprehensibility and feasibility, the main study started in 2012 when 10,580 postal questionnaires were sent out. In the following 5970 residential addresses had to be searched via local registration offices.

### Disease status

Two of the authors extracted the diagnosis made at the first admission and the date of the first admission to the GCPAR from the medical records. As information on the onset of disease was not available in one third of the patients, the date of the first admission to the GCPAR was calculated as a proxy for disease duration.

As the definition of childhood chronic arthritis was changed several times since 1952 [20, 21] we had to re-diagnose patients treated before 1997, the year when the current definition of JIA was put into place. This procedure has formerly been performed in order to evaluate the ILAR criteria for JIA [22].

Therefore the definition of JIA followed established criteria:

- Patients diagnosed before 1978 were re-classified according to the ILAR-classification based on their medical recordings [*N* = 433 (11.7%)] [23] This included patients diagnosed with Spondylarthitis, Psoriatic Arthritis, Rheumatoid Arthritis or Still’s disease (with an onset before the age of 16 years, disease duration of at least 6 weeks and no other cause of symptoms)
- JCA-patients diagnosed between 1978 and 1996 according the classification criteria of the European League Against Rheumatism [*N* = 1290 (34.9%)] [24]
- JIA-patients diagnosed after 1996 had been classified based on the classification criteria of the International League of Associations for Rheumatology [*N* = 1975 (53.4%)] [20, 25]

### Questionnaire

In 2012, data was collected via a self-administered standardized questionnaire, containing validated questions of the ISAAC [26] and the ECRHS [27] study on the following aspects:

- Socio-demographics: sex; age; participants’ and parental education; employment status

Disease related questions were newly designed and face-validated by two experts:

Disease related data: holding a disability card (yes/no - For the determination of the requirement of a disability card, there are nationwide guidelines, which contain general assessment rules on the disease specific determination of the degree of disability [28]; current intake of anti-rheumatic drugs (yes/no - the medication classified as anti-rheumatic drugs included cytostatics, glucocorticoids, immunosuppressives, DMARDs, biologic agents); current treatment (yes/no).

### Variable definition

#### Participants’ educational level

The highest school degree of the participants was available from the questionnaire in the following categories: Primary School (“Hauptschule/Volksschule”), Secondary School (“Mittlere Reife/Realschule”), College (“Abitur/Fachabitur”), University (“Hochschule/Fachhochschule/Universität”), other degree.

In Germany the average age for the attendance of Primary School ranges from 5 to 18 years, for Secondary School and Collage from 8 to 18 years and for University the average entrance date is 21 years [29, 30].

#### Parental education

Highest level of schooling achieved by the parents was used to define parental level of education applying the same coding scheme as for the participants’ educational level. Information on the parents’ highest educational degree was available for 1801 participants [Missing values: *n* = 382 (21%)].

For both groups we categorized the educational achievements into “*Primary School Degree”*, *“Secondary School Degree”* and *“College or University Degree”*, in order to differentiate between low, medium and high level of education [31]. Participants that indicated “Other degree” were excluded as no definite level of schooling can be determined.

#### Employment

All participants indicating full- or at least part-time employment were categorized as being employed. Participants who indicated to carry out a profession less than 8 h per day or less than 40 h a week were categorized as being part-time employed.

### Data management and statistical analyses

#### Eligible study population

**Fig. 1:**
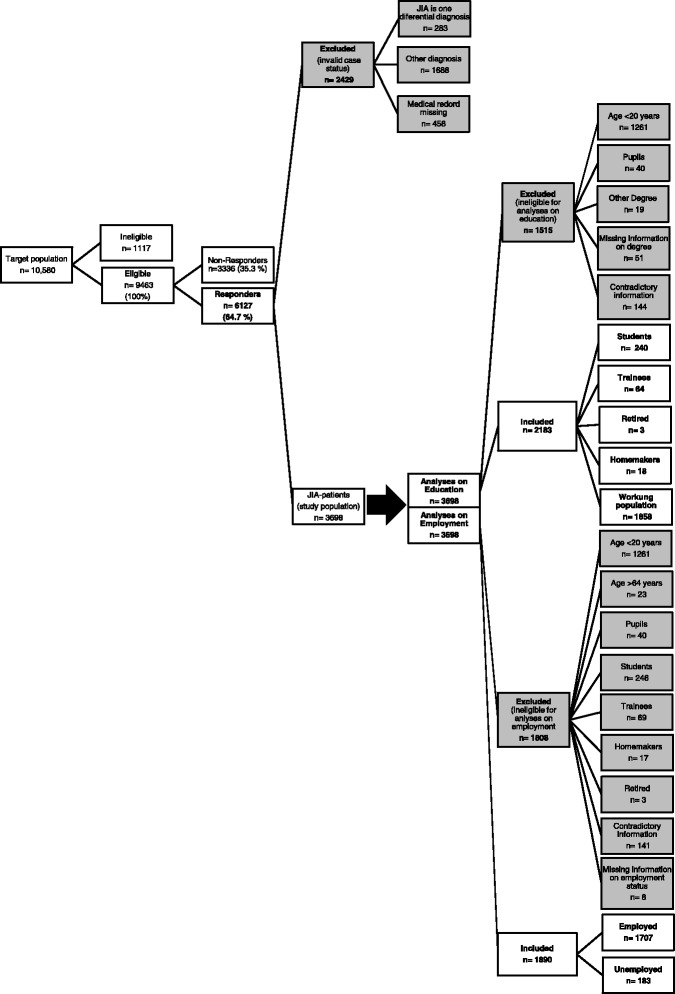
Response and inclusion criteria of the study population. Eligibility criteria for the analyses on educational achievement and employment

Statistical analyses using level of education as outcome needed to be restricted to JIA-patients older than 19 years (*n* = 2696) due to the restrictions of the National Educational Panel Study (NEPS). Therefore, JIA-patients were excluded (Fig. 1) if:

- aged under 20 years [32],
- not graduate from school (pupils) yet
- no clear assignment was possible to the group of pupils or already graduated persons (students, trainees, working population, homemakers, retired) due to contradictory information within the questionnaire. (e.g. participants indicated a highest school degree but mentioned in the questionnaire that they were currently attending further education and that they worked part-time)
- missing information on educational degree

For the analyses of unemployment participants were excluded (Fig. 1) if:

- younger than 20 years or older than 64 years. As the national data on the employment status of the German general population [33] only considers the working population between 20 and 64;
- data classify them to be a pupil, student, trainee, homemaker or retired subject as profession
- no clear classification was possible to the group of pupils or already graduated persons (students, trainees, working population, homemakers, retired) due to contradictory information within the questionnaire. (e.g. participants indicated a school degree, but noted within the questionnaire that they were currently doing further education and that they worked part-time)
- employment status was missing.

#### Data entry

Double-entry of data and congruence checking was performed using Surveymonkey database (Surveymonkey Inc., USA) and Synkronizer Version 10.0 (©2000-2014, XL Consulting GmbH, Schweiz).

#### Statistical analyses

SPSS program version 22.0 (IBM Corporation, USA) was used for the statistical analyses.

For the description of socio-demographic and disease related characteristics absolute and relative frequencies were calculated. For age and disease duration, measures of the central tendency (mean, median) and measures of dispersion (range, standard deviation) were calculated.

Patients’ and parental level of education were directly sex- and age-standardized to the NEPS-dataset for the general population [32]. For this, we stratified age (20–29; 30-39; 40–49; 50–59; ≥60 yrs) and sex according to the standard population. Subsequently the age- and sex-standardized relative differences were calculated with 95% confidence interval between the study population and the general population. Using the age categories provided by the Federal Statistical Office Germany (20–34; 35–49; 50–64 yrs) the same method was applied for employment status [34].

For sensitivity analyses the age- and sex-standardized educational degree and employment status were stratified by year of diagnoses. This was done as changes in treatment might have improved educational achievements and employment possibilities of JIA-patients [34]. For this two time periods, diagnoses made before 2001 or after 2000 were compared. This was done as biologics were approved for treatment of JIA in Germany around the year 2000 [35]. This change was of great clinical significance, since biologics alleviate symptoms rapidly and can be used in patients who did not respond on conventional therapies [36, 37]. In addition, within both time periods, stratification was performed for patients that were still under treatment due to their rheumatic disease and those who were not. Small age strata (*n* < 10) within the study population were excluded from analyses as they may produce invalid results [38].

As 31% of the SEPIA study population was from the Bavarian part of Germany, which has the lowest unemployment rate in Germany [39], a separate standardization of the Bavarian JIA-patients to the Bavarian general population (BGP) was conducted.

## Results

The response rate was 66% with 6127 filled out questionnaires returned and of these, 3698 (60%) were identified as JIA-patients. The excluded patients (*n* = 2429) suffered from other (rheumatic) diseases. A total of 2183 fulfilled the inclusion criteria for the analyses about their own as well as the parental level of education. For the analyses on employment status, 1890 JIA-patients were eligible (Fig. 1).

### Descriptive data

Table 1 shows the non-standardized distribution of socio-demographic characteristics of the SEPIA study population and the GGP. A significantly higher proportion of JIA-patients were female in comparison to the general population. In addition, JIA-patients were on average more than 10 years younger than the GGP. About half of the JIA-patients reported taking anti-rheumatic drugs at the time of the survey (Table 1). Over one third of the study population were disability cardholders. The median disease duration approximated by the date of first admission to the hospital was 20 years with a wide range (1 to 60 years).

**Table 1:** Comparison of socio-demographic and disease related characteristics of the SEPIA study population and the GGP

	SEPIA study population		German general population^a^	
Total	*N* = 2183	n_missing_	*N* = 11,799	n_missing_
Sex				
Female	60.8% (*n* = 1327)	0	50.8% (*n* = 6065)	0
Age (years)				
Mean (± SD^b^)	34.4 (± 10.8)	0	46.9 (± 11.2)	10
Median (Range)	32.0 (20.0 – 73.0)		47.9 (20.3 – 67.5)	
Current intake of anti-rheumatic drugs	49.0% (*n* = 1068)	0	N.a.^c^	
Disability cardholder	37.3% (*n* = 815)	0	N.a.^c^	
Disease duration^d^				
Mean (± SD^b^) Median (Range)	22.8 (± 11.6) 20.0 (1.0 - 60.0)	0	N.a.^c^	

^a^NEPS Data

^b^SD = Standard Deviation

^c^N.a. = Not available

^d^Disease duration approximated by the date of the 1^st^ admission to the GCPAR

### Educational degree of the JIA-patients and the German general population

The non-standardized distribution of the educational degree of the JIA-patients indicated a higher level of schooling than in GGP with 51% of the JIA-patients having achieved a high educational degree as compared to 44% in GGP (Table 2). However, after age- and sex-standardization, 3% more of the JIA-patients had only achieved the lowest level of education (95% Confidence Interval: 1.91 to 4.12%) while medium and high educational achievements did not differ statistically significant.

**Table 2:** Age- and sex-standardized comparison of the educational degree of the study population and the GGP

				Primary School Degree			Secondary School Degree			College or University Degree		
	Age groups^a^ [years]	N GGP^b^	N Sepia	GGP^b^ n (%)	SEPIA n (%)	Standardized SEPIA n (%) ^c^	GGP^b^ n (%)	SEPIA n (%)	Standardized SEPIA n (%)^c^	GGP^b^ n (%)	SEPIA n (%)	Standardized SEPIA n (%)^c^
Men	20–29	624	306	99 (15.87)	55 (17.97)	112 (17.97)	170 (27.24)	95 (31.05)	194 (31.05)	355 (56.89)	156 (50.98)	318 (50.98)
	30–39	772	230	157 (20.34)	50 (21.74)	168 (21.74)	209 (27.07)	69 (30.00)	232 (30.00)	406 (52.59)	111 (48.26)	373 (48.26)
	40–49	1542	231	348 (22.57)	52 (22.51)	347 (22.51)	501 (32.49)	69 (29.87)	461 (29.87)	693 (44.94)	110 (47.62)	734 (47.62)
	50–59	2052	59	553 (26.95)	14 (23.73)	487 (23.73)	555 (27.05)	19 (32.20)	661 (32.20)	944 (46.00)	26 (44.07)	904 (44.07)
	≥60	812	30	359 (44.21)	13 (43.33)	352 (43.33)	177 (21.80)	8 (26.67)	217 (26.67)	276 (33.99)	9 (30.00)	244 (30.00)
Women	20–29	482	618	35 (7.26)	61 (9.87)	48 (9.87)	136 (28.22)	201 (32.52)	157 (32.52)	311 (64.52)	356 (57.61)	278 (57.61)
	30–39	820	344	118 (14.39)	36 (10.47)	86 (10.47)	285 (34.76)	110 (31.98)	262 (31.98)	417 (50.85)	198 (57.56)	472 (57.56)
	40–49	1753	242	227 (12.95)	37 (15.29)	268 (15.29)	786 (44.84)	97 (40.08)	703 (40.08)	740 (42.21)	108 (44.63)	782 (44.63)
	50–59	2182	97	538 (24.66)	40 (41.24)	900 (41.24)	847 (38.82)	28 (28.87)	630 (28.87)	797 (36.53)	29 (29.90)	652 (29.90)
	≥60	740	26	290 (39.19)	11 (42.31)	313 (42.31)	240 (32.43)	8 (30.77)	228 (30.77)	210 (28.38)	7 (26.92)	199 (26.92)
Total		11,789	2183	2724 (23.11)	369 (16.90)	3080 (26.13)	3906 (33.13)	704 (32.25)	3742 (31.75)	5149 (43.68)	1110 (50.85)	4956 (42.04)
Standardized Difference %^d^ (95% CI)^e^						3.02 (1.91; 4.12)			−1.39 (−2.59; −0.19)			−1.63 (−2.91; −0.37)

^a^Categorization of age groups was set by NEPS dataset of the German general population

^b^GGP = German general population [Ref.]

^c^Standardized proportion of the SEPIA study population

^d^Standardized Difference (%) = Standardized Proportion of SEPIA (%) – Proportion of GGP (%)

^e^95% CI = 95% Confidence Interval

### Sensitivity analyses

#### JIA-patients admitted to the GCPAR before 2001

Stratifying for the date of admission (treated **before the year 2001** (*n* = 1887)) a 3% (95% CI: 1.94 to 4.15%) higher proportion of patients were shown to have a lower level of education as compared to the GGP. The differences in medium and high education remained non-significant. In patients still **under treatment** (*n* = 922) no statistically significant difference in the education was found as compared to the general population. Among patients who indicated **not being under treatment** at time of the survey (*n* = 962) 5% more than in GGP achieved only a primary school degree (3.34 to 5.57%) and 4% less than the general population reached a secondary school degree (−5.18 to −2.18%). There was no statistically significant difference between the proportions of highly educated persons in both groups. (Additional file 1).

#### JIA-patients admitted to the GCPAR after 2000

Comparing JIA-patients admitted **after 2000** (*n* = 288) to the German general population revealed no significant differences. When stratifying for current treatment status 5% (2.09 to 8.11%) more of participants that indicated to be still **in need of treatment** were less educated than the general population. Furthermore, there was no statistical significant difference in the educational achievements of JIA-patients and the German general population for those admitted after the year 2000 and **not receiving treatment** at the time of the survey (Additional file 1).

### Standardized comparison of the parental education of the SEPIA study population and the German general population

Educational level of participants’ parents was almost equally distributed between low, medium and high level of education (Table 3). Comparing the standardized educational achievement to the general population, JIA-patients’ parents were equally likely to have only reached primary education, while they were more likely to hold a *“Secondary School Degree”* (difference 3%; 95% CI: 2.31 to 4.37%) and less frequently held *“College or University Degree”* (−3%; −4.21 to −2.03%).

**Table 3:** Age- and sex-standardized comparison of the parental education within the study population and the GGP

				Primary School Degree			Secondary School Degree			College or University Degree		
	Age groups^a^ [years]	N GGP^b^	N Sepia	GGP^b^ n (%)	SEPIA n (%)	Standardized SEPIA n (%)^c^	GGP^b^ n (%)	SEPIA n (%)	Standardized SEPIA n (%)^c^	GGP^b^ n (%)	SEPIA n (%)	Standardized SEPIA n (%)^c^
Men	20–29	618	257	153 (24.76)	52 (20.23)	125 (20.23)	179 (28.96)	93 (36.19)	224 (36.19)	286 (46.28)	112 (43.58)	269 (43.58)
	30–39	767	192	303 (39.50)	77 (40.10)	308 (40.10)	197 (25.68)	58 (30.21)	232 (30.21)	267 (34.81)	57 (29.69)	228 (29.69)
	40–49	1527	180	907 (59.40)	113 (62.78)	959 (62.78)	240 (15.72)	47 (26.11)	399 (26.11)	380 (24.89)	20 (11.11)	170 (11.11)
	50–59	2053	50	1336 (65.08)	33 (66.00)	1355 (66.00)	301 (14.66)	11 (22.00)	452 (22.00)	416 (20.26)	6 (12.00)	246 (12.00)
	> = 60	809	22	592 (73.18)	16 (72.73)	588 (72.73)	98 (12.11)	3 (13.64)	110 (13.64)	119 (14.71)	3 (13.64)	110 (13.64)
Women	20–29	481	532	117 (24.32)	105 (19.74)	95 (19.74)	158 (32.85)	190 (35.71)	172 (35.71)	206 (42.83)	237 (44.55)	214 (44.55)
	30–39	818	277	331 (40.46)	100 (36.10)	295 (36.10)	219 (26.77)	68 (24.55)	201 (24.55)	268 (32.76)	109 (39.35)	322 (39.35)
	40–49	1729	195	981 (56.74)	109 (55.90)	967 (55.90)	333 (19.26)	37 (18.97)	328 (18.97)	415 (24.00)	49 (25.13)	435 (25.13)
	50–59	2179	79	1399 (64.20)	52 (65.82)	1434 (65.82)	323 (14.82)	12 (15.19)	331 (15.19)	457 (20.97)	15 (18.99)	414 (18.99)
	> = 60	747	17	515 (68.94)	11 (64.71)	483 (64.71)	96 (12.85)	2 (11.76)	88 (11.76)	136 (18.21)	4 (23.53)	176 (23.53)
Total		11,728	1801	6634 (56.57)	668 (37.09)	6609 (56.35)	2144 (18.28)	521 (28.93)	2536 (21.62)	2950 (25.15)	612 (33.98)	2584 (22.03)
Standardized Difference %^d^ (95% CI)^e^						−0.21 (−1.49; 1.06)			3.34 (2.31; 4.37)			−3.12 (−4.21; −2.03)

^a^Categorization of age groups was set by NEPS dataset of the German general population

^b^GGP = German general population [Ref.]

^c^Standardized proportion of the SEPIA study population

^d^Standardized Difference (%) = Standardized Proportion of SEPIA (%) – Proportion of GGP (%)

^e^95% CI = 95% Confidence Interval

***Unemployment among the SEPIA study population and the German general population.***

For JIA-patients, a non-standardized unemployment of 9.7% was observed which went up to 12.9% after direct age- and sex-standardization to GGP. The 0.2% difference compared to the GGP was statistically significant (95% CI 0.16 to 0.19%) (Table 4).

**Table 4:** Age- and sex-standardized comparison of the unemployment rate of the study population and the GGP

				Unemployment		
	Age groups^a^ [years]	N GGP^b^	N Sepia	GGP^b^ n (%)	SEPIA n (%)	Standardized SEPIA n (%)^c^
Men	20–34	6,135,000	342	427,000 (6.96)	13 (3.80)	233,202 (3.80)
	35–49	8,431,000	355	381,000 (4.52)	20 (5.63)	474,986 (5.63)
	50–64	9,904,000	74	3,680,000 (37.16)	11 (14.86)	1,472,216 (14.86)
Women	20–34	5,335,000	616	312,000 (5.85)	36 (5.84)	311,786 (5.84)
	35–49	7,336,000	391	336,000 (4.58)	69 (17.65)	1,294,588 (17.65)
	50–64	5,650,000	112	291,000 (5.13)	34 (30.36)	1,715,178 (30.36)
Total		42,791,000	1890	5,427,000 (12.68)	183 (9.68)	5,501,956 (12.86)
Standardized Difference %^d^ (95% CI)^e^						0.18 (0.16; 0.19)

^a^Categorization of age groups was set by the data on employment of the German General population of the Federal Statistical Office Germany

^b^GGP = German general population [Ref.]

^c^Standardized proportion of the SEPIA study population

^d^Standardized Difference (%) = Standardized Proportion of SEPIA (%) – Proportion of GGP (%)

^e^95% CI = 95% Confidence Interval

### Sensitivity analyses

#### JIA-patients admitted to the GCPAR before 2001

In sensitivity analyses a standardized difference of 0.1% (95% CI: 0.07 to 0.10%) indicated that the proportion of unemployed JIA-patients **admitted before 2001** (*n* = 1691) was almost equal to that of the general population. However, those still **receiving treatment** due to JIA were more likely to be unemployed (6%; 6.27 to 6.30%) while those **not requiring treatment** were statistically significantly less likely to be unemployed (−4%; −4.23 to −4.21%). (Additional file 2).

#### JIA-patients admitted to the GCPAR after 2000

Comparing JIA-patients admitted after 2000 to the general population, a 0.5% (−0.45 to −0.49%) lower standardized proportion of unemployment was observed (*n* = 195). Again, this differed by treatment status: Of the patients still **reliant on treatment** (*n* = 126) the proportion of unemployment was 1.5% (1.47 to 1.51%) higher, among those **not requiring treatment** it was 2% (−2.29 to −2.25%) lower than in the general population. (Additional file 2).

#### Subgroup of Bavarian JIA-patients

The Bavarian study population had in total a 3% higher proportion of unemployment (95% CI: 3.27 to 3.34%) than the Bavarian general population.

**Patients admitted to the GCPAR before 2001** were significantly more likely to be unemployed (3%; 3.12 to 3.28%) when restricting the analyses to the Bavarian sub-cohort**.** In patients still **reliant on treatment** the unemployment was 8% (7.42 to 7.49%) above that of the BGP. However, in those who did **not require treatment** at the time of the survey, the unemployment was almost equal (−0.1%; −0.17 to −0.11%) to that of the BGP.

The standardized difference in Bavarian JIA-patients **admitted to the GCPAR after 2000** remained comparable to that of the total study population (−0.5%; −0.53 to −0.42%). Patients’ still **receiving treatment** at the time of the survey had a 4% (4.04 to 4.16%) higher unemployment rate than in the BGP. Patients that were **not reliant on treatment** any more were statistically significantly less likely (−4%; −4.29 to −4.20%) to be unemployed than the general population. (Additional file 3).

## Discussion

In the present study, JIA-patients had on average achieved a lower level of education and were slightly but statistically significantly more likely to be unemployed than the GGP. Distinctions in parental level of education could not explain this difference indicating that this might be a consequence of their disease.

Our findings are in line with some but not all previous studies. E.g., Packham et al. reported that JIA-patients from the UK were better educated while their unemployment rate was twice as high compared to the control population. They investigated 246 JIA-patients of which 72% were female, the mean age was 35 years, the patients siblings served as controls [8]. Foster and colleagues, however, did not observe a difference in the educational attainment of JIA-patients compared to local controls in the UK. They included 82 patients of which 83% were female, the median age was 30 years and the local population severed as controls [4]. In line with our results Bouaddi et al. found that the schooling of Moroccan children with JIA was negatively impacted by their chronic illness. They included 33 JIA-patients and 74 healthy controls, 46% of the observed patients were female with a median age of 11 years [3]. Within the SEPIA study we investigated the educational achievements of 2183 JIA-patients of which 61% were female; the median age was 32 years. The age- and sex-standardized comparison to the GGP showed that JIA-patients received statistical significantly lower educational degrees. Although within Germany the number of young people entering university is constantly increasing [40], the proportion of students suffering from health restrictions remained around 7% (*n* = 137,000) within the time range from 2000 to 2012 [41]. One explanation for poor educational performance might be increased absenteeism-rates within pupils that suffer from chronic diseases [3, 42–44]. Bouaddi et al. observed that JIA-patients were 3-times more often absent then the healthy controls and 33% were not even able to attend school [3]. Moreover, patients with chronic conditions were found to have more days of absences than could be attributed to their chronic disease [43]. Confirming these findings a study by Moonie et al. reported a negative impact of absenteeism on the test performance for all participants comparing the educational achievements of asthma patients to their non-asthmatic peers [44]. In accordance with these findings adolescents with chronic conditions were found to have higher school dropout rates [43].

Interpreting these inconsistencies between studies, differences in health- [45] and education systems as well as the social insurance systems have to be taken into account. In Germany, 90% of the pupils attend public schools, [46] and 93% of all students are enrolled at a public university [47]. In addition, about 86% of the German population are members of public health insurances [48], which covers e.g. the JIA treatment costs. Therefore, the disease might not have a large impact on families’ income as it could be the case in other countries where families have to cover costs for treatment and education.

In Germany, since 2002, programs supporting employers to hire persons with disability which offer an extended protection from termination, contribute a part of the salary and offer financial support for better accessibility are in place [49]. Additionally entrepreneurs with more than 20 employers are legally obligated to employ at least 5% disabled persons [50]. Despite disability legislations we showed a higher overall rate of unemployment in JIA-patients compared to the GGP. These differences were mainly due to higher likelihood of unemployment in patients with active disease. Sensitivity analyses further showed that the unemployment in participants admitted to the GCPAR after the year 2000 was 0.5% lower than in the GGP. This circumstance could on one hand be due to the already mentioned disability legislations; on the other hand they might be a result of better treatment options. Due to progress in early detection and holistic treatment options better patient outcomes could be achieved [36, 37, 51]. The increased unemployment rates within the Bavarian sub-set are due to Bavaria being a rather prosperous region of Germany where the unemployment is on average lower [39, 52].

The recruitment of a large sample of JIA-patients followed from childhood until adulthood is the main strength of the study. Furthermore, data was collected via a detailed questionnaire containing validated standardized questions. As only questionnaire data on the highest school degree, sex (categorical items) and age (calculated via date of birth) was considered, we do not assume major reporting bias. Additionally, direct standardization enabled an unbiased comparison to the GGP.

Some limitations of our study have to be taken into account as well. As the National Disability Program and the use of biologic drugs were introduced in early 2000, young patients might be more likely to have experienced the beneficial effects of the improved access to employment and new treatment possibilities. However, future studies might be more likely to show the beneficial effects of these measures as many of the participants admitted after 2000 were still under the age of 20 years and had therefore been excluded from our analyses. Furthermore, we were not able to conduct analyses on the non-responders, as no data was available. This group of patients might contain more severe cases, which were not able to answer the questionnaire because of their health condition. In addition, on the basis of the available data we were not able to consider the impact of the disease on delaying the graduation or the entry into the work force. No further analyses on patients classified as students, trainees, homeworkers or retired persons were conducted because no data on the background of their employment status was available, although it might be associated to disease related factors. It is also due to a lack of data, that we had to approximate the disease duration by the date of the first admission to the GCPAR. This might lead to biased results since the delay between the onset of symptoms and the first admission could be highly variable. However, we choose this approach as it rather underestimates the effect of disease duration.

Although a response of 66% can be considered as reasonably high in an epidemiological study of this kind, one still has to take selection bias into account. In general, persons with a higher level of education are more likely to participate in studies [53–55]. This would have led to an underestimation of effect, i.e., the true level of education among JIA-patients would even be lower than in our study. Generalizability of our results to all JIA-patients might be limited by the fact that we recruited JIA-patients from a specialized hospital where more severe cases are likely to be treated.

## Conclusion

In conclusion, our findings indicate that especially JIA-patients with longer disease duration or active disease still have a lower educational attainment and higher unemployment rate than the general population. This calls for further improvement of treatment options in order to minimize the permanent impact of JIA on patients’ life. Further progress in maintaining mobility and effective pain management could facilitate the access to higher educational degrees and a successful profession. Furthermore, more supporting programs within the education system are necessary to increase the chance on higher educational qualifications. Occupational therapy interventions could improve the entry into the working environment.

## Additional files

Additional file 1: Table S1. Age- and sex-standardized comparison of the educational achievements of the study population admitted to the GCPAR before 2001 and the GGP. (DOCX 49 kb)

Additional file 2: Table S2. Crude and age and sex-standardized comparison of the employment status of the SEPIA study population admitted to the GCPAR before 2001 and the German General Population. (DOCX 32 kb)

Additional file 3: Table S3. Crude and age- and sex-standardized comparison of the unemployment rate of the SEPIA study population and the *Bavarian General Population. (DOCX 38 kb)*

## References

[1] Malviya A, Rushton SP, Foster HE, Ferris CM, Hanson H, Muthumayandi K (2012). The relationships between adult juvenile idiopathic Arthritis and employment. Arthritis Rheum.

[2] Ravelli A, Martini A (2007). Juvenile idiopathic arthritis. Lancet.

[3] Bouaddi I, Rostom S, El Badri D, Hassani A, Chkirate B, Amine B (2013). Impact of juvenile idiopathic arthritis on schooling. BMC Pediatr.

[4] Foster HE, Marshall N, Myers A, Dunkley P, Griffiths ID (2003). Outcome in adults with juvenile idiopathic arthritis: a quality of life study. Arthritis Rheum.

[5] Eisenstein EM, Berkun Y. Diagnosis and classification of juvenile idiopathic arthritis. J Autoimmun. 2014;(48-49):31–3. 10.1016/j.jaut.2014.01.009 24461383

[6] Minden K, Niewerth M, Listing J, Mobius D, Thon A, Ganser G (2009). The economic burden of juvenile idiopathic arthritis-results from the German paediatric rheumatologic database. Clin Exp Rheumatol.

[7] Gerhardt CA, McGoron KD, Vannatta K, McNamara KA, Taylor J, Passo M (2008). Educational and occupational outcomes among young adults with juvenile idiopathic Arthritis. Arthritis Rheumatism Arthritis Care Res.

[8] Packham JC, Hall MA (2002). Long-term follow-up of 246 adults with juvenile idiopathic arthritis: social function, relationships and sexual activity. Rheumatology.

[9] Minden K, Niewerth M, Listing J, Biedermann T, Bollow M, Schontube M (2002). Long-term outcome in patients with juvenile idiopathic arthritis. Arthritis Rheum.

[10] Miller JJ (1993). Psychosocial factors related to rheumatic diseases in childhood. J Rheumatol Suppl..

[11] White PH, Shear ES (1992). Transition/job readiness for adolescents with juvenile arthritis and other chronic illness. J Rheumatol Suppl.

[12] Archenholtz B, Nordborg E, Bremell T (2001). Lower level of education in young adults with arthritis starting in the early adulthood. Scand J Rheumatol.

[13] Peterson LS, Mason T, Nelson AM, O'Fallon WM, Gabriel SE (1997). Psychosocial outcomes and health status of adults who have had juvenile rheumatoid arthritis - a controlled, population-based study. Arthritis Rheum.

[14] Flato B, Lien GH, Smerdel A, Vinje O, Dale K, Johnston V (2003). Prognostic factors in juvenile rheumatoid arthritis: a case-control study revealing early predictors and outcome after 14.9 years. J Rheumatol.

[15] Diaz-Mendoza AC, Modesto Caballero C, Navarro-Cendejas J (2015). Analysis of employment rate and social status in young adults with childhood-onset rheumatic disease in Catalonia. Pediatr Rheumatol Online J..

[16] Jetha A (2015). The impact of arthritis on the early employment experiences of young adults: a literature review. Disabil Health J.

[17] Gerhardt CA, McGoron KD, Vannatta K, McNamara KA, Taylor J, Passo M (2008). Educational and occupational outcomes among young adults with juvenile idiopathic arthritis. Arthritis Rheum.

[18] Jetha A, Badley E, Beaton D, Fortin PR, Shiff NJ, Gignac MA (2015). Unpacking early work experiences of young adults with rheumatic disease: an examination of absenteeism, job disruptions, and productivity loss. Arthritis Care Res (Hoboken).

[19] Fischer M, Geis W (2013). Bestimmungsgrößen der Bildungsmobilität in Deutschland. IW-Trends : Vierteljahresschrift zur empirischen Wirtschaftsforschung aus dem Institut der Deutschen Wirtschaft Köln.

[20] Duffy CM, Colbert RA, Laxer RM, Schanberg LE, Bowyer SL (2005). Nomenclature and classification in chronic childhood arthritis - time for a change?. Arthritis Rheum.

[21] Petty RE, Southwood TR, Manners P, Baum J, Glass DN, Goldenberg J (2004). International league of associations for rheumatology classification of juvenile idiopathic arthritis: second revision, Edmonton, 2001. J Rheumatol.

[22] Krumrey-Langkammerer M, Hafner R (2001). Evaluation of the ILAR criteria for juvenile idiopathic arthritis. J Rheumatol.

[23] Brewer EJ, Bass J, Baum J, Cassidy JT, Fink C, Jacobs J (1977). Current proposed revision of JRA criteria. JRA criteria Subcommittee of the Diagnostic and Therapeutic Criteria Committee of the American rheumatism section of the Arthritis Foundation. Arthritis Rheum.

[24] Wood P (1978). Special meeting on nomenclature and classification of arthritis in children. The care of rheumatic children [internet].

[25] Petty RE (1998). Classification of childhood arthritis: a work in progress. Bailliere Clin Rheum.

[26] Weiland SK, Bjorksten B, Brunekreef B, Cookson WO, von Mutius E, Strachan DP (2004). Phase II of the international study of asthma and allergies in childhood (ISAAC II): rationale and methods. Eur Respir J.

[27] European Community Respiratory Health survey. Available from: http://www.ecrhs.org/Quests/ECRHSIImainquestionnaire.pdf. Accessed 15 May 2017.

[28] Verbraucherschutz BfJu. Verordnung zur Durchführung des § 1 Abs. 1 und 3, des § 30 Abs. 1 und des § 35 Abs. 1 des Bundesversorgungsgesetzes (Versorgungsmedizin-Verordnung - VersMedV) Anlage zu § 2 der Versorgungsmedizin-Verordnung vom 10. Dezember 2008 2008. Available from: http://www.gesetze-im-internet.de/versmedv/anlage.html. Accessed 15 May 2017.

[29] Entwicklung des Durchschnittsalters von Studierenden und Absolventen an deutschen Hochschulen seit 2000. 2008. https://www.destatis.de/DE/Publikationen/WirtschaftStatistik/BildungForschungKultur/DurchschnittsalterStudierende.pdf?__blob=publicationFile. Accessed 15 May 2017.

[30] Bildung und Kultur (2015). Allgemeinbildende Schulen. Schuljahr 2014/2015.

[31] Jöckel K-H, Babitsch B, Bellach B-M, Bloomfield K, Hoffmeyer-Zlotnik J, Winkler J (1998). Messung und Quantifizierung soziodemographischer Merkmale in epidemiologischen Studien. Messung Soziodemographischer Merkmale Epidemiologie RKI-Schriften.

[32] Blossfeld H-P, von Maurice J (2011). Education as a lifelong process. Z Erziehungswiss.

[33] Mikrozensus Bevölkerung und Erwerbstätigkeit: Stand und Entwicklung der Erwerbstätigkeit in Deutschland. https://www.destatis.de/DE/Publikationen/Thematisch/Arbeitsmarkt/Erwerbstaetige/StandEntwicklungErwerbstaetigkeit2010411127004.pdf?__blob=publicationFile. Accessed 15 May 2017

[34] Minden K, Niewerth M, Zink A, Seipelt E, Foeldvari I, Girschick H (2012). Long-term outcome of patients with JIA treated with etanercept, results of the biologic register JuMBO. Rheumatology.

[35] Horneff G, Forster J, Seyberth HW, Michels H, Arbeitgemeinschaft Kinder- und J. Recommendations by the pediatric and adolescent rheumatology study committee on therapy with Etanercept (p75 TNF-alpha receptor immunoglobulin fusion protein. Pharmacotherapy committee Z Rheumatol 2000;59(6):365-9. 10.1007/s003930070043 11201000

[36] Hashkes PJ, Uziel Y, Laxer RM (2010). The safety profile of biologic therapies for juvenile idiopathic arthritis. Nat Rev Rheumatol.

[37] Klein A, Horneff G (2009). Treatment strategies for juvenile idiopathic arthritis. Expert Opin Pharmacother.

[38] Curtin LR, Klein RJ, Statistics NCfH. Direct standardization (age-adjusted death rates). Hyattsville: U.S. Department of Health and Human Services, Public Health Service, Centers for Disease Control and Prevention, National Center for Health Statistics; 1995.

[39] Durchschnittliche Arbeitslosenquote nach Bundesländern im Jahr 2014: statista; 2014. Available from: http://de.statista.com/statistik/daten/studie/2192/umfrage/durchschnittliche-arbeitslosenquote-nach-bundeslaendern/. Accessed 15 May 2017.

[40] Wachsender Studentenberg – Entwicklung der Studierendenzahlen in Deutschland: Bundeszentrale für Politische Bildung; 2014. Available from: http://www.bpb.de/gesellschaft/kultur/zukunft-bildung/190350/wachsender-studentenberg-entwicklung-der-studierendenzahlen-in-deutschland. Accessed 15 May 2017.

[41] Middendorff EABPJKMNN. Die wirtschaftliche und soziale Lage der Studierenden in Deutschland 2012 - 20. Sozialerhebung des Deutschen Studentenwerks durchgeführt durch das HIS-Institut für Hochschulforschung: Bundesministerium für Bildung und Forschung; 2012. Available from: https://www.studentenwerke.de/sites/default/files/01_20-SE-Hauptbericht.pdf. Accessed 15 May 2017.

[42] Sturge C, Garralda M, Boissin M, Dore C, Woo P (1997). School attendance and juvenile chronic arthritis. Br J Rheumatol.

[43] Suris JC, Michaud PA, Viner R (2004). The adolescent with a chronic condition. Part I: developmental issues. Arch Dis Child.

[44] Moonie S, Sterling D, Figgs L, Castro M (2008). The relationship between school absence, academic performance, and asthma status. J Sch Health.

[45] Hugle B, Haas JP, Benseler SM (2013). Treatment preferences in juvenile idiopathic arthritis - a comparative analysis in two health care systems. Pediatr Rheumatol Online J.

[46] Statistisches Bundesamt W (2012). Schulen auf einen Blick.

[47] Statistisches Bundesamt W (2014). Bildung und Kultur.

[48] Statistisches Bundesamt W (2012). Angaben zur Krankenversicherung - (Ergebnisse der Mikrozensus 2011).

[49] Behindertengleichstellungsgesetze. Available from: http://www.behindertenbeauftragte.de/DE/Themen/RechtlicheGrundlagen/Behindertengleichstellungsgesetz/Behindertengleichstellungsgesetz_node.html. Accessed 15 May 2017.

[50] Allgemeines Gleichbehandlungsgesetz (AGG) (2006). Bundesministerium der Justiz und Verbraucherschutz.

[51] Davies R, Gaynor D, Hyrich KL, Pain CE. Efficacy of biologic therapy across individual juvenile idiopathic arthritis subtypes: a systematic review. Semin Arthritis Rheum. 2016; 10.1016/j.semarthrit.2016.10.008 27914689

[52] Nettoeinkommen und verfügbares Nettoeinkommen pivater Haushalte im Monat nach Bundesländern Essen: statista; 2015. Available from: http://de.statista.com/statistik/daten/studie/5758/umfrage/verfuegbares-nettoeinkommen-nach-bundeslaendern/. Accessed 15 May 2017.

[53] Sonne-Holm S, Sorensen TI, Jensen G, Schnohr P (1989). Influence of fatness, intelligence, education and sociodemographic factors on response rate in a health survey. J Epidemiol Community Health.

[54] Korkeila K, Suominen S, Ahvenainen J, Ojanlatva A, Rautava P, Helenius H (2001). Non-response and related factors in a nation-wide health survey. Eur J Epidemiol.

[55] Langhammer A, Krokstad S, Romundstad P, Heggland J, Holmen J (2012). The HUNT study: participation is associated with survival and depends on socioeconomic status, diseases and symptoms. BMC Med Res Methodol.

