# Supplementary material for: Education and employment in patients with juvenile idiopathic arthritis – a standardized comparison to the German general population
Source: Pediatr Rheumatol Online J. 2017 May 22;15:45. doi: 10.1186/s12969-017-0172-2 (PMC5440947; doi:10.1186/s12969-017-0172-2)
Supplement: Supplementary file 1 — Age- and sex-standardized comparison of the educational achievements of the study population admitted to the GCPAR before 2001 and the GGP. (DOCX 49 kb) [file 12969_2017_172_MOESM1_ESM.docx]

**Additional File 1: Education**

Table S1: Age- and sex-standardized comparison of the educational achievements of the study population admitted to the GCPAR before 2001 and the GGP

|  | | | | **Primary School Degree** | | | **Secondary School Degree** | | | **College or University Degree** | | |
| --- | --- | --- | --- | --- | --- | --- | --- | --- | --- | --- | --- | --- |
|  | Age groups^1^  [years] | **N GGP^2^** | **N Sepia** | **GGP^2^**  **n (%)** | **SEPIA**  **n (%)** | **Standardized**  **SEPIA**  **n (%)^3^** | **GGP^2^**  **n (%)** | **SEPIA**  **n (%)** | **Standardized**  **SEPIA**  **n (%)^3^** | **GGP^2^**  **n (%)** | **SEPIA**  **n (%)** | **Standardized**  **SEPIA**  **n (%)^3^** |
| **Men** | 20-29 | 624 | 216 | 99 (15.87) | 39 (18.06) | 113 (18.06) | 170 (27.24) | 74 (34.26) | 214 (34.26) | 355 (56.89) | 103 (47.69) | 298 (47.69) |
|  | 30-39 | 772 | 229 | 157 (20.34) | 50 (21.83) | 169 (21.83) | 209 (27.07) | 69 (30.13) | 233 (30.13) | 406 (52.59) | 110 (48.03) | 371 (48.03) |
|  | 40-49 | 1542 | 231 | 348 (22.57) | 52 (22.51) | 347 (22.51) | 501 (32.49) | 69 (29.87) | 461 (29.87) | 693 (44.94) | 110 (47.62) | 734 (47.62) |
|  | 50-59 | 2052 | 59 | 553 (26.95) | 14 (23.73) | 487 (23.73) | 555 (27.05) | 19 (32.20) | 661 (32.20) | 944 (46.00) | 26 (44.07) | 904 (44.07) |
|  | ≥60 | 812 | 30 | 359 (44.21) | 13 (43.33) | 352 (43.33) | 177 (21.80) | 8 (26.67) | 217 (26.67) | 276 (33.99) | 9 (30.00) | 244 (30.00) |
| **Women** | 20-29 | 482 | 420 | 35 (7.26) | 45 (10.71) | 52 (10.71) | 136 (28.22) | 123 (29.29) | 141 (29.29) | 311 (64.52) | 252 (60.00) | 289 (60.00) |
|  | 30-39 | 820 | 337 | 118 (14.39) | 34 (10.09) | 83 (10.09) | 285 (34.76) | 108 (32.05) | 263 (32.05) | 417 (50.85) | 195 (57.86) | 475 (57.86) |
|  | 40-49 | 1753 | 242 | 227 (12.95) | 37 (15.29) | 268 (15.29) | 786 (44.84) | 97 (40.08) | 703 (40.08) | 740 (42.21) | 108 (44.63) | 782 (44.63) |
|  | 50-59 | 2182 | 97 | 538 (24.66) | 40 (41.24) | 900 (41.24) | 847 (38.82) | 28 (28.87) | 630 (28.87) | 797 (36.53) | 29 (29.90) | 652 (29.90) |
|  | ≥60 | 740 | 26 | 290 (39.19) | 11 (42.31) | 313 (42.31) | 240 (32.43) | 8 (30.77) | 228 (30.77) | 210 (28.38) | 7 (26.92) | 199 (26.92) |
| **Total** |  | **11779** | **1887** | **2724 (23.13)** | **335 (17.75)** | **3083 (26.17)** | **3906 (33.16)** | **603 (31.96)** | **3749 (31.82)** | **5149 (43.71)** | **949 (50.29)** | **4948 (42.01)** |
| **Stand.**  **Dif. %^4^**  **(95% CI)^5^** |  |  |  |  |  | **3.04**  **(1.94; 4.15)** |  |  | **-1.34**  **(-2.54; -0.14)** |  |  | **-1.71**  **(-2.97; -0.44)** |

^1^ Categorization of age groups was set by NEPS dataset of the German general population

^2^ GGP= German general population [Ref.]

^3^ Standardized proportion of the SEPIA study population

^4^ Standardized Difference (%)= Standardized Proportion of SEPIA (%) – Proportion of GGP (%)

^5^ 95% CI= 95% Confidence Interval

Table A1.1: Age- and sex-standardized comparison of the educational achievements of the study population admitted to the GCPAR before 2001 still reliant on treatment and the GGP

|  | | | | **Primary School Degree** | | | **Secondary School Degree** | | | **College or University Degree** | | |
| --- | --- | --- | --- | --- | --- | --- | --- | --- | --- | --- | --- | --- |
|  | Age groups^1^  [years] | **N GGP^2^** | **N Sepia** | **GGP^2^**  **n (%)** | **SEPIA**  **n (%)** | **Standardized**  **SEPIA**  **n (%)^3^** | **GGP^2^**  **n (%)** | **SEPIA**  **n (%)** | **Standardized**  **SEPIA**  **n (%)^3^** | **GGP^2^**  **n (%)** | **SEPIA**  **n (%)** | **Standardized**  **SEPIA**  **n (%)^3^** |
| **Men** | 20-29 | 624 | 80 | 99 (15.87) | 13 (16.25) | 101 (16.25) | 170 (27.24) | 31 (38.75) | 242 (38.75) | 355 (56.89) | 36 (45.00) | 281 (45.00) |
|  | 30-39 | 772 | 82 | 157 (20.34) | 21 (25.61) | 198 (25.61) | 209 (27.07) | 26 (31.71) | 245 (31.71) | 406 (52.59) | 35 (42.68) | 330 (42.68) |
|  | 40-49 | 1542 | 82 | 348 (22.57) | 17 (20.73) | 320 (20.73) | 501 (32.49) | 28 (34.15) | 527 (34.15) | 693 (44.94) | 37 (45.12) | 696 (45.12) |
|  | 50-59 | 2052 | 18 | 553 (26.95) | 4 (22.22) | 456 (22.22) | 555 (27.05) | 3 (16.67) | 342 (16.67) | 944 (46.00) | 11 (61.11) | 1254 (61.11) |
|  | ≥60 | 812 | 10 | 359 (44.21) | 2 (20.00) | 162 (20.00) | 177 (21.80) | 5 (50.00) | 406 (50.00) | 276 (33.99) | 3 (30.00) | 244 (30.00) |
| **Women** | 20-29 | 482 | 251 | 35 (7.26) | 28 (11.16) | 54 (11.16) | 136 (28.22) | 78 (31.08) | 150 (31.08) | 311 (64.52) | 145 (57.77) | 279 (57.77) |
|  | 30-39 | 820 | 205 | 118 (14.39) | 27 (13.17) | 108 (13.17) | 285 (34.76) | 65 (31.71) | 260 (31.71) | 417 (50.85) | 113 (55.12) | 452 (55.12) |
|  | 40-49 | 1753 | 135 | 227 (12.95) | 21 (15.56) | 273 (15.56) | 786 (44.84) | 54 (40.00) | 701 (40.00) | 740 (42.21) | 60 (44.44) | 779 (44.44) |
|  | 50-59 | 2182 | 49 | 538 (24.66) | 17 (34.69) | 757 (34.69) | 847 (38.82) | 20 (40.82) | 891 (40.82) | 797 (36.53) | 12 (24.49) | 534 (24.49) |
|  | ≥60 | 740 | 10 | 290 (39.19) | 4 (40.00) | 296 (40.00) | 240 (32.43) | 4 (40.00) | 296 (40.00) | 210 (28.38) | 2 (20.00) | 148 (20.00) |
| **Total** |  | **11779** | **922** | **2724 (23.13)** | **154 (16.70)** | **2725 (23.13)** | **3906 (33.16)** | **314 (34.06)** | **4059 (34.46)** | **5149 (43.71)** | **454 (49.24)** | **4996 (42.41)** |
| **Stand.**  **Dif. %^4^**  **(95% CI)^5^** |  |  |  |  |  | **0.01**  **(-1.09; 1.08)** |  |  | **1.30**  **(0.08; 2.51)** |  |  | **-1.30**  **(-2.57; -0.03)** |

^1^ Categorization of age groups was set by NEPS dataset of the German general population

^2^ GGP= German general population [Ref.]

^3^ Standardized proportion of the SEPIA study population

^4^ Standardized Difference (%)= Standardized Proportion of SEPIA (%) – Proportion of GGP (%)

^5^ 95% CI= 95% Confidence Interval

Table A1.2: Age- and sex-standardized comparison of the educational achievements of the SEPIA study population admitted to the GCPAR before 2001 not reliant on treatment and the GGP

|  | | | | **Primary School Degree** | | | **Secondary School Degree** | | | **College or University Degree** | | |
| --- | --- | --- | --- | --- | --- | --- | --- | --- | --- | --- | --- | --- |
|  | Age groups^1^  [years] | **N GGP^2^** | **N Sepia** | **GGP^2^**  **n (%)** | **SEPIA**  **n (%)** | **Standardized**  **SEPIA**  **n (%)^3^** | **GGP^2^**  **n (%)** | **SEPIA**  **n (%)** | **Standardized**  **SEPIA**  **n (%)^3^** | **GGP^2^**  **n (%)** | **SEPIA**  **n (%)** | **Standardized**  **SEPIA**  **n (%)^3^** |
| **Men** | 20-29 | 624 | 136 | 99 (15.87) | 26 (19.12) | 119 (19.12) | 170 (27.24) | 43 (31.62) | 197 (31.62) | 355 (56.89) | 67 (49.26) | 307 (49.26) |
|  | 30-39 | 772 | 147 | 157 (20.34) | 29 (19.73) | 152 (19.73) | 209 (27.07) | 43 (29.25) | 226 (29.25) | 406 (52.59) | 75 (51.02) | 394 (51.02) |
|  | 40-49 | 1542 | 149 | 348 (22.57) | 35 (23.49) | 362 (23.49) | 501 (32.49) | 41 (27.52) | 424 (27.52) | 693 (44.94) | 73 (48.99) | 756 (48.99) |
|  | 50-59 | 2052 | 40 | 553 (26.95) | 9 (22.50) | 462 (22.50) | 555 (27.05) | 16 (40.00) | 821 (40.00) | 944 (46.00) | 15 (37.50) | 770 (37.50) |
|  | ≥60 | 812 | 19 | 359 (44.21) | 10 (52.63) | 427 (52.63) | 177 (21.80) | 3 (15.79) | 128 (15.79) | 276 (33.99) | 6 (31.58) | 256 (31.58) |
| **Women** | 20-29 | 482 | 169 | 35 (7.26) | 17 (10.06) | 49 (10.06) | 136 (28.22) | 45 (26.63) | 128 (26.63) | 311 (64.52) | 107 (63.31) | 305 (63.31) |
|  | 30-39 | 820 | 132 | 118 (14.39) | 7 (5.30) | 44 (5.30) | 285 (34.76) | 43 (32.58) | 267 (32.58) | 417 (50.85) | 82 (62.12) | 509 (62.12) |
|  | 40-49 | 1753 | 106 | 227 (12.95) | 16 (15.09) | 265 (15.09) | 786 (44.84) | 42 (39.62) | 695 (39.62) | 740 (42.21) | 48 (45.28) | 794 (45.28) |
|  | 50-59 | 2182 | 48 | 538 (24.66) | 23 (47.92) | 1046 (47.92) | 847 (38.82) | 8 (16.67) | 364 (16.67) | 797 (36.53) | 17 (35.42) | 773 (35.42) |
|  | ≥60 | 740 | 16 | 290 (39.19) | 7 (43.75) | 324 (43.75) | 240 (32.43) | 4 (25.00) | 185 (25.00) | 210 (28.38) | 5 (31.25) | 231 (31.25) |
| **Total** |  | **11779** | **962** | **2724 (23.13)** | **179 (18.61)** | **3249 (27.58)** | **3906 (33.16)** | **288 (29.94)** | **3435 (29.16)** | **5149 (43.71)** | **495 (51.46)** | **5095 (43.26)** |
| **Stand.**  **Dif. %^4^**  **(95% CI)^5^** |  |  |  |  |  | **4.45**  **(3.34; 5.57)** |  |  | **-4.00**  **(-5.18; -2.18)** |  |  | **-0.46**  **(-1.73; 0.81)** |

^1^ Categorization of age groups was set by NEPS dataset of the German general population

^2^ GGP= German general population [Ref.]

^3^ Standardized proportion of the SEPIA study population

^4^ Standardized Difference (%)= Standardized Proportion of SEPIA (%) – Proportion of GGP (%)

^5^ 95% CI= 95% Confidence Interval

Table A2: Age- and sex-standardized comparison of the educational achievements of the SEPIA study population admitted to the GCPAR after 2000 and the GGP

|  | | | | **Primary School Degree** | | | **Secondary School Degree** | | | **College or University Degree** | | |
| --- | --- | --- | --- | --- | --- | --- | --- | --- | --- | --- | --- | --- |
|  | Age groups^1^  [years] | **N GGP^2^** | **N Sepia** | **GGP^2^**  **n (%)** | **SEPIA**  **n (%)** | **Standardized**  **SEPIA**  **n (%)^3^** | **GGP^2^**  **n (%)** | **SEPIA**  **n (%)** | **Standardized**  **SEPIA**  **n (%)^3^** | **GGP^2^**  **n (%)** | **SEPIA**  **n (%)** | **Standardized**  **SEPIA**  **n (%)^3^** |
| **Men** | 20-29 | 624 | 90 | 99 (15.87) | 16 (17.78) | 111 (17.78) | 170 (27.24) | 21 (23.33) | 146 (23.33) | 355 (56.89) | 53 (58.89) | 368 (58.89) |
| **Women** | 20-29 | 482 | 198 | 35 (7.26) | 16 (8.08) | 39 (8.08) | 136 (28.22) | 78 (39.39) | 190 (39.39) | 311 (64.52) | 104 (52.53) | 253 (52.53) |
| **Total** |  | **1106** | **288** | **134 (12.12)** | **32 (11.11)** | **150 (13.55)** | **306 (27.67)** | **99 (34.38)** | **336 (34.38)** | **666 (60.22)** | **157 (54.51)** | **621 (56.21)** |
| **Stand.**  **Dif. %^4^**  **(95% CI)^5^** |  |  |  |  |  | **1.44**  **(-1.42; 4.29)** |  |  | **2.67**  **(-1.18; 6.50)** |  |  | **-4.10**  **(-8.26; 0.07)** |

^1^ Categorization of age groups was set by NEPS dataset of the German general population

^2^ GGP= German general population [Ref.]

^3^ Standardized proportion of the SEPIA study population

^4^ Standardized Difference (%)= Standardized Proportion of SEPIA (%) – Proportion of GGP (%)

^5^ 95% CI= 95% Confidence Interval

Table A2.1: Age- and sex-standardized comparison of the educational achievements of the SEPIA study population admitted to the GCPAR after 2000 still reliant on treatment and the GGP

|  | | | | **Primary School Degree** | | | **Secondary School Degree** | | | **College or University Degree** | | |
| --- | --- | --- | --- | --- | --- | --- | --- | --- | --- | --- | --- | --- |
|  | Age groups^1^  [years] | **N GGP^2^** | **N Sepia** | **GGP^2^**  **n (%)** | **SEPIA**  **n (%)** | **Standardized**  **SEPIA**  **n (%)^3^** | **GGP^2^**  **n (%)** | **SEPIA**  **n (%)** | **Standardized**  **SEPIA**  **n (%)^3^** | **GGP^2^**  **n (%)** | **SEPIA**  **n (%)** | **Standardized**  **SEPIA**  **n (%)^3^** |
| **Men** | 20-29 | 624 | 45 | 99 (15.87) | 11 (24.44) | 153 (24.44) | 170 (27.24) | 8 (17.78) | 111 (17.78) | 355 (56.89) | 26 (57.78) | 361 (57.78) |
| **Women** | 20-29 | 482 | 140 | 35 (7.26) | 11 (7.86) | 38 (7.86) | 136 (28.22) | 57 (40.71) | 196 (40.71) | 311 (64.52) | 72 (51.43) | 248 (51.43) |
| **Total** |  | **1106** | **185** | **134 (12.12)** | **22 (11.89)** | **190 (17.22)** | **306 (27.67)** | **65 (35.14)** | **307 (27.77)** | **666 (60.22)** | **98 (52.97)** | **608 (55.01)** |
| **Stand.**  **Dif. %^4^**  **(95% CI)^5^** |  |  |  |  |  | **5.10**  **(2.09; 8.11)** |  |  | **0.11**  **(-3.68; 3.90)** |  |  | **-5.21**  **(-9.36; -1.02)** |

^1^ Categorization of age groups was set by NEPS dataset of the German general population

^2^ GGP= German general population [Ref.]

^3^ Standardized proportion of the SEPIA study population

^4^ Standardized Difference (%)= Standardized Proportion of SEPIA (%) – Proportion of GGP (%)

^5^ 95% CI= 95% Confidence Interval

Table A2.2: Age- and sex-standardized comparison of the educational achievements of the SEPIA study population admitted to the GCPAR after 2000 not reliant on treatment and the GGP

|  | | | | **Primary School Degree** | | | **Secondary School Degree** | | | **College or University Degree** | | |
| --- | --- | --- | --- | --- | --- | --- | --- | --- | --- | --- | --- | --- |
|  | Age groups^1^  [years] | **N GGP^2^** | **N Sepia** | **GGP^2^**  **n (%)** | **SEPIA**  **n (%)** | **Standardized**  **SEPIA**  **n (%)^3^** | **GGP^2^**  **n (%)** | **SEPIA**  **n (%)** | **Standardized**  **SEPIA**  **n (%)^3^** | **GGP^2^**  **n (%)** | **SEPIA**  **n (%)** | **Standardized**  **SEPIA**  **n (%)^3^** |
| **Men** | 20-29 | 624 | 45 | 99 (15.87) | 5 (11.11) | 69 (11.11) | 170 (27.24) | 13 (28.89) | 180 (28.89) | 355 (56.89) | 27 (60.00) | 374 (60.00) |
| **Women** | 20-29 | 482 | 58 | 35 (7.26) | 5 (8.62) | 42 (8.62) | 136 (28.22) | 21 (36.21) | 175 (36.21) | 311 (64.52) | 32 (55.17) | 266 (55.17) |
| **Total** |  | **1106** | **103** | **134 (12.12)** | **10 (9.71)** | **111 (10.03)** | **306 (27.67)** | **34 (33.01)** | **355 (32.08)** | **666 (60.22)** | **59 (57.28)** | **640 (57.90)** |
| **Stand.**  **Dif. %^4^**  **(95% CI)^5^** |  |  |  |  |  | **-2.09**  **(-4.78; 0.60)** |  |  | **4.41**  **(0.53; 8.27)** |  |  | **-2.32**  **(-6.47; 1.84)** |

^1^ Categorization of age groups was set by NEPS dataset of the German general population

^2^ GGP= German general population [Ref.]

^3^ Standardized proportion of the SEPIA study population

^4^ Standardized Difference (%)= Standardized Proportion of SEPIA (%) – Proportion of GGP (%)

^5^ 95% CI= 95% Confidence Interval
