# Supplementary material for: Education and employment in patients with juvenile idiopathic arthritis – a standardized comparison to the German general population
Source: Pediatr Rheumatol Online J. 2017 May 22;15:45. doi: 10.1186/s12969-017-0172-2 (PMC5440947; doi:10.1186/s12969-017-0172-2)
Supplement: Supplementary file 2 — Crude and age and sex-standardized comparison of the employment status of the SEPIA study population admitted to the GCPAR before 2001 and the German General Population. (DOCX 32 kb) [file 12969_2017_172_MOESM2_ESM.docx]

**Additional file 2: Employment**

Table S2: Crude and age and sex-standardized comparison of the employment status of the SEPIA study population admitted to the GCPAR before 2001 and the German General Population

|  | | | | **Unemployment** | | |
| --- | --- | --- | --- | --- | --- | --- |
|  | Age groups^1^  [years] | **N GGP^2^** | **N Sepia** | **GGP^2^**  **n (%)** | **SEPIA**  **n (%)** | **Standardized**  **SEPIA**  **n (%)^3^** |
| **Men** | 20-34 | 6,135,000 | 282 | 427,000 (6.96) | 9 (3.19) | 195,798 (3.19) |
|  | 35-49 | 8,431,000 | 353 | 381,000 (4.52) | 20 (5.67) | 477,677 (5.67) |
|  | 50-64 | 9,904,000 | 74 | 3,680,000 (37.16) | 11 (14.86) | 1,472,216 (14.86) |
| **Women** | 20-34 | 5,335,000 | 481 | 312,000 (5.85) | 29 (6.03) | 321,653 (6.03) |
|  | 35-49 | 7,336,000 | 389 | 336,000 (4.58) | 68 (17.48) | 1,282,386 (17.48) |
|  | 50-64 | 5,650,000 | 112 | 291,000 (5.13) | 34 (30.36) | 1,715,177 (30.36) |
| **Total** |  | **42,791,000** | **1691** | **5,427,000 (12.68)** | **171 (10.11)** | **5,464,908 (12.77)** |
| **Stand.**  **Dif. %^4^**  **(95% CI)^5^** |  |  |  |  |  | **0.09**  **(0.07; 0.10)** |

^1^ Categorization of age groups was set by the data on employment of the German General population of the Federal Statistical Office Germany

^2^ GGP= German general population

^3^ Standardized proportion of the SEPIA study population

^4^ Standardized Difference (%)= Standardized Proportion of SEPIA (%) – Proportion of GGP (%)

^5^ 95% CI= 95% Confidence Interval

Table B1.1: Crude and age and sex-standardized comparison of the employment status of the SEPIA study population admitted to the GCPAR before 2001 still reliant on treatment and the German General Population

|  | | | | **Unemployment** | | |
| --- | --- | --- | --- | --- | --- | --- |
|  | Age groups^1^  [years] | **N GGP^2^** | **N Sepia** | **GGP^2^**  **n (%)** | **SEPIA**  **n (%)** | **Standardized**  **SEPIA**  **n (%)^3^** |
| **Men** | 20-34 | 6,135,000 | 103 | 427,000 (6.96) | 7 (6.80) | 416,942 (6.80) |
|  | 35-49 | 8,431,000 | 121 | 381,000 (4.52) | 14 (11.57) | 975,488 (11.57) |
|  | 50-64 | 9,904,000 | 21 | 3,680,000 (37.16) | 5 (23.81) | 2,358,095 (23.81) |
| **Women** | 20-34 | 5,335,000 | 283 | 312,000 (5.85) | 13 (4.59) | 245,071 (4.59) |
|  | 35-49 | 7,336,000 | 226 | 336,000 (4.58) | 51 (22.57) | 1,655,469 (22.57) |
|  | 50-64 | 5,650,000 | 55 | 291,000 (5.13) | 24 (43.64) | 2,465,455 (43.64) |
| **Total** |  | **42,791,000** | **809** | **5,427,000 (12.68)** | **114 (14.09)** | **8,116,519 (18.97)** |
| **Stand.**  **Dif. %^4^**  **(95% CI)^5^** |  |  |  |  |  | **6.29**  **(6.27; 6.30)** |

^1^ Categorization of age groups was set by the data on employment of the German General population of the Federal Statistical Office Germany

^2^ GGP= German general population

^3^ Standardized proportion of the SEPIA study population

^4^ Standardized Difference (%)= Standardized Proportion of SEPIA (%) – Proportion of GGP (%)

^5^ 95% CI= 95% Confidence Interval

Table B1.2: Crude and age and sex-standardized comparison of the employment status of the SEPIA study population admitted to the GCPAR before 2001 not reliant on treatment and the German General Population

|  | | | | **Unemployment** | | |
| --- | --- | --- | --- | --- | --- | --- |
|  | Age groups^1^  [years] | **N GGP^2^** | **N Sepia** | **GGP^2^**  **n (%)** | **SEPIA**  **n (%)** | **Standardized**  **SEPIA**  **n (%)^3^** |
| **Men** | 20-34 | 6,135,000 | 179 | 427,000 (6.96) | 2 (1.12) | 68,548 (1.12) |
|  | 35-49 | 8,431,000 | 232 | 381,000 (4.52) | 6 (2.59) | 218,043 (2.59) |
|  | 50-64 | 9,904,000 | 52 | 3,680,000 (37.16) | 6 (11.54) | 1,142,769 (11.54) |
| **Women** | 20-34 | 5,335,000 | 198 | 312,000 (5.85) | 16 (8.08) | 431,111 (8.08) |
|  | 35-49 | 7,336,000 | 162 | 336,000 (4.58) | 17 (10.49) | 769,827 (10.49) |
|  | 50-64 | 5,650,000 | 57 | 291,000 (5.13) | 10 (17.54) | 991,228 (17.54) |
| **Total** |  | **42,791,000** | **880** | **5,427,000 (12.68)** | **57 (6.48)** | **3,621,526 (8.46)** |
| **Stand.**  **Dif. %^4^**  **(95% CI)^5^** |  |  |  |  |  | **-4.22**  **(-4.23; -4.21)** |

^1^ Categorization of age groups was set by the data on employment of the German General population of the Federal Statistical Office Germany

^2^ GGP= German general population

^3^ Standardized proportion of the SEPIA study population

^4^ Standardized Difference (%)= Standardized Proportion of SEPIA (%) – Proportion of GGP (%)

^5^ 95% CI= 95% Confidence Interval

Table B2: Crude and age and sex-standardized comparison of the employment status of the SEPIA study population admitted to the GCPAR after 2000 and the German General Population

|  | | | | **Unemployment** | | |
| --- | --- | --- | --- | --- | --- | --- |
|  | Age groups^1^  [years] | **N GGP^2^** | **N Sepia** | **GGP^2^**  **n (%)** | **SEPIA**  **n (%)** | **Standardized**  **SEPIA**  **n (%)^3^** |
| **Men** | 20-34 | 6,135,000 | 60 | 427,000 (6.96) | 4 (6.67) | 409,000 (6.67) |
| **Women** | 20-34 | 5,335,000 | 135 | 312,000 (5.85) | 7 (5.19) | 276,630 (5.19) |
| **Total** |  | **11,470,000** | **195** | **739,000 (6.44)** | **11 (5.64)** | **685,630 (5.98)** |
| **Stand.**  **Dif. %^4^**  **(95% CI)^5^** |  |  |  |  |  | **-0.47**  **(-0.45; - 0.49)** |

^1^ Categorization of age groups was set by the data on employment of the German General population of the Federal Statistical Office Germany

^2^ GGP= German general population

^3^ Standardized proportion of the SEPIA study population

^4^ Standardized Difference (%)= Standardized Proportion of SEPIA (%) – Proportion of GGP (%)

^5^ 95% CI= 95% Confidence Interval

Table B2.1: Crude and age and sex-standardized comparison of the employment status of the SEPIA study population admitted to the GCPAR after 2000 still reliant on treatment and the German General Population

|  | | | | **Unemployment** | | |
| --- | --- | --- | --- | --- | --- | --- |
|  | Age groups^1^  [years] | **N GGP^2^** | **N Sepia** | **GGP^2^**  **n (%)** | **SEPIA**  **n (%)** | **Standardized**  **SEPIA**  **n (%)^3^** |
| **Men** | 20-34 | 6,135,000 | 29 | 427,000 (6.96) | 3 (10.34) | 634,655 (10.34) |
| **Women** | 20-34 | 5,335,000 | 97 | 312,000 (5.85) | 5 (5.15) | 275,000 (5.15) |
| **Total** |  | **11,470,000** | **126** | **739,000 (6.44)** | **8 (6.35)** | **909,655 (7.93)** |
| **Stand.**  **Dif. %^4^**  **(95% CI)^5^** |  |  |  |  |  | **1.49**  **(1.47; 1.51)** |

^1^ Categorization of age groups was set by the data on employment of the German General population of the Federal Statistical Office Germany

^2^ GGP= German general population

^3^ Standardized proportion of the SEPIA study population

^4^ Standardized Difference (%)= Standardized Proportion of SEPIA (%) – Proportion of GGP (%)

^5^ 95% CI= 95% Confidence Interval

Table B2.2: Crude and age and sex-standardized comparison of the employment status of the SEPIA study population admitted to the GCPAR after 2000 not reliant on treatment and the German General Population

|  | | | | **Unemployment** | | |
| --- | --- | --- | --- | --- | --- | --- |
|  | Age groups^1^  [years] | **N GGP^2^** | **N Sepia** | **GGP^2^**  **n (%)** | **SEPIA**  **n (%)** | **Standardized**  **SEPIA**  **n (%)^3^** |
| **Men** | 20-34 | 6,135,000 | 31 | 427,000 (6.96) | 1 (3.23) | 197,903 (3.23) |
| **Women** | 20-34 | 5,335,000 | 38 | 312,000 (5.85) | 2 (5.26) | 280,790 (5.26) |
| **Total** |  | **11,470,000** | **69** | **739,000 (6.44)** | **3 (4.35)** | **478,693 (4.17)** |
| **Stand.**  **Dif. %^4^**  **(95% CI)^5^** |  |  |  |  |  | **-2.27**  **(-2.29; -2.25)** |

^1^ Categorization of age groups was set by the data on employment of the German General population of the Federal Statistical Office Germany

^2^ GGP= German general population

^3^ Standardized proportion of the SEPIA study population

^4^ Standardized Difference (%)= Standardized Proportion of SEPIA (%) – Proportion of GGP (%)

^5^ 95% CI= 95% Confidence Interval
