# Supplementary material for: Education and employment in patients with juvenile idiopathic arthritis – a standardized comparison to the German general population
Source: Pediatr Rheumatol Online J. 2017 May 22;15:45. doi: 10.1186/s12969-017-0172-2 (PMC5440947; doi:10.1186/s12969-017-0172-2)
Supplement: Supplementary file 3 — Crude and age- and sex-standardized comparison of the unemployment rate of the SEPIA study population and the Bavarian General Population. (DOCX 38 kb) [file 12969_2017_172_MOESM3_ESM.docx]

**Additional file 3: Employment within the Bavarian sub-cohort**

Table S3: Crude and age- and sex-standardized comparison of the unemployment rate of the SEPIA study population and the *Bavarian General Population*

|  | | | | **Unemployment** | | |
| --- | --- | --- | --- | --- | --- | --- |
|  | Age groups^1^  [years] | **N BGP^2^** | **N Sepia** | **BGP^2^**  **n (%)** | **SEPIA**  **n (%)** | **Standardized**  **SEPIA**  **n (%)^3^** |
| **Men** | 20-34 | 995,000 | 118 | 72,000 (7.24) | 5 (4.24) | 42,161 (4.24) |
|  | 35-49 | 1,508,000 | 104 | 88,000 (5.84) | 5 (4.81) | 72,500 (4.81) |
|  | 50-64 | 872,000 | 13 | 74,000 (8.49) | 1 (7.69) | 67,077 (7.69) |
| **Women** | 20-34 | 861,000 | 218 | 73,000 (8.48) | 14 (6.42) | 55294 (6.42) |
|  | 35-49 | 1,243,000 | 104 | 87,000 (7.00) | 17 (16.35) | 203,183 (16.35) |
|  | 50-64 | 676,000 | 24 | 68,000 (8.20) | 8 (33.33) | 225,333 (33.33) |
| **Total** |  | **6,155,000** | **581** | **462,000** | **50** | **665,548** |
| **Stand.**  **Dif. %^4^**  **(95% CI)^5^** |  |  |  |  |  | **3.31**  **(3.27; 3.34)** |

^1^ Categorization of age groups was set by the data on employment of the German General population of the Federal Statistical Office Germany

^2^ BGP= Bavarian general population

^3^ Standardized proportion of the SEPIA study population

^4^ Standardized Difference (%)= Standardized Proportion of SEPIA (%) – Proportion of BGP (%)

^5^ 95% CI= 95% Confidence Interval

TableB3.1: Crude and age and sex-standardized comparison of the employment status of the SEPIA study population admitted to the GCPAR before 2001 and the *Bavarian General Population*

|  | | | | **Unemployment** | | |
| --- | --- | --- | --- | --- | --- | --- |
|  | Age groups^1^  [years] | **N BGP^2^** | **N Sepia** | **BGP^2^**  **n (%)** | **SEPIA**  **n (%)** | **Standardized**  **SEPIA**  **n (%)^3^** |
| **Men** | 20-34 | 995,000 | 89 | 72,000 (7.24) | 3 (3.37) | 33,539 (3.37) |
|  | 35-49 | 1,508,000 | 103 | 88,000 (5.84) | 5 (4.85) | 73,204 (4.85) |
|  | 50-64 | 872,000 | 13 | 74,000 (8.49) | 1 (7.69) | 67,077 (7.69) |
| **Women** | 20-34 | 861,000 | 167 | 73,000 (8.48) | 10 (5.99) | 51,557 (5.99) |
|  | 35-49 | 1,243,000 | 103 | 87,000 (7.00) | 17 (16.50) | 205,155 (16.50) |
|  | 50-64 | 676,000 | 24 | 68,000 (8.20) | 8 (33.33) | 225,333 (33.33) |
| **Total** |  | **6,155,000** | **499** | **462,000** | **44** | **655,866** |
| **Stand.**  **Dif. %^4^**  **(95% CI)^5^** |  |  |  |  |  | **3.15**  **(3.12; 3.18)** |

^1^ Categorization of age groups was set by the data on employment of the German General population of the Federal Statistical Office Germany

^2^ BGP= Bavarian general population

^3^ Standardized proportion of the SEPIA study population

^4^ Standardized Difference (%)= Standardized Proportion of SEPIA (%) – Proportion of BGP (%)

^5^ 95% CI= 95% Confidence Interval

Table B3.1.1: Crude and age and sex-standardized comparison of the employment status of the SEPIA study population admitted to the GCPAR before 2001 still reliant on treatment and *the Bavarian General Population*

|  | | | | **Unemployment** | | |
| --- | --- | --- | --- | --- | --- | --- |
|  | Age groups^1^  [years] | **N BGP^2^** | **N Sepia** | **BGP^2^**  **n (%)** | **SEPIA**  **n (%)** | **Standardized**  **SEPIA**  **n (%)^3^** |
| **Men** | 20-34 | 995,000 | 29 | 72,000 (7.24) | 2 (6.90) | 68,621 (6.90) |
|  | 35-49 | 1,508,000 | 30 | 88,000 (5.84) | 3 (10.00) | 150,800 (10.00) |
| **Women** | 20-34 | 861,000 | 85 | 73,000 (8.48) | 3 (3.53) | 30,388 (3.53) |
|  | 35-49 | 1,243,000 | 57 | 87,000 (7.00) | 12 (21.05) | 261,684 (21.05) |
|  | 50-64 | 676,000 | 10 | 68,000 (8.20) | 4 (40.00) | 270,400 (40.00) |
| **Total** |  | **5,283,000** | **211** | **388,000** | **24** | **781,893** |
| **Stand.**  **Dif. %^4^**  **(95% CI)^5^** |  |  |  |  |  | **7.46**  **(7.42; 7.49)** |

^1^ Categorization of age groups was set by the data on employment of the German General population of the Federal Statistical Office Germany

^2^ BGP= Bavarian general population

^3^ Standardized proportion of the SEPIA study population

^4^ Standardized Difference (%)= Standardized Proportion of SEPIA (%) – Proportion of BGP (%)

^5^ 95% CI= 95% Confidence Interval

Table B3.1.2 Crude and age and sex-standardized comparison of the employment status of the SEPIA study population admitted to the GCPAR before 2001 not reliant on treatment and the *Bavarian General Population*

|  | | | | **Unemployment** | | |
| --- | --- | --- | --- | --- | --- | --- |
|  | Age groups^1^  [years] | **N BGP^2^** | **N Sepia** | **BGP^2^**  **n (%)** | **SEPIA**  **n (%)** | **Standardized**  **SEPIA**  **n (%)^3^** |
| **Men** | 20-34 | 995,000 | 60 | 72,000 (7.24) | 1 (1.67) | 16,583 (1.67) |
|  | 35-49 | 1,508,000 | 73 | 88,000 (5.84) | 2 (2.74) | 41,315 (2.74) |
|  | 50-64 | 872,000 | 10 | 74,000 (8.49) | 1 (10.00) | 87,200 (10.00) |
| **Women** | 20-34 | 861,000 | 82 | 73,000 (8.48) | 7 (8.54) | 73,500 (8.54) |
|  | 35-49 | 1,243,000 | 45 | 87,000 (7.00) | 5 (11.11) | 138,111 (11.11) |
|  | 50-64 | 676,000 | 14 | 68,000 (8.20) | 2 (14.29) | 96,571 (14.29) |
| **Total** |  | **6,155,000** | **284** | **462,000** | **18** | **453,281** |
| **Stand.**  **Dif. %^4^**  **(95% CI)^5^** |  |  |  |  |  | **-0.14**  **(-0.17; -0.11)** |

^1^ Categorization of age groups was set by the data on employment of the German General population of the Federal Statistical Office Germany

^2^ BGP= Bavarian general population

^3^ Standardized proportion of the SEPIA study population

^4^ Standardized Difference (%)= Standardized Proportion of SEPIA (%) – Proportion of BGP (%)

^5^ 95% CI= 95% Confidence Interval

TableB3.2: Crude and age and sex-standardized comparison of the employment status of the SEPIA study population admitted to the GCPAR after 2000 and the *Bavarian General Population*

|  | | | | **Unemployment** | | |
| --- | --- | --- | --- | --- | --- | --- |
|  | Age groups^1^  [years] | **N BGP^2^** | **N Sepia** | **BGP^2^**  **n (%)** | **SEPIA**  **n (%)** | **Standardized**  **SEPIA**  **n (%)^3^** |
| **Men** | 20-34 | 995,000 | 29 | 72,000 (7.24) | 2 (6.90) | 68,621 (6.90) |
| **Women** | 20-34 | 861,000 | 51 | 73,000 (8.48) | 4 (7.84) | 67,529 (7.84) |
| **Total** |  | **1,856,000** | **80** | **145,000** | **6** | **136,150** |
| **Stand.**  **Dif. %^4^**  **(95% CI)^5^** |  |  |  |  |  | **-0.48**  **(-0.53; -0.42)** |

^1^ Categorization of age groups was set by the data on employment of the German General population of the Federal Statistical Office Germany

^2^ BGP= Bavarian general population

^3^ Standardized proportion of the SEPIA study population

^4^ Standardized Difference (%)= Standardized Proportion of SEPIA (%) – Proportion of BGP (%)

^5^ 95% CI= 95% Confidence Interval

Table B3.2.1: Crude and age and sex-standardized comparison of the employment status of the SEPIA study population admitted to the GCPAR after 2000 still reliant on treatment and *the Bavarian General Population*

|  | | | | **Unemployment** | | |
| --- | --- | --- | --- | --- | --- | --- |
|  | Age groups^1^  [years] | **N BGP^2^** | **N Sepia** | **BGP^2^**  **n (%)** | **SEPIA**  **n (%)** | **Standardized**  **SEPIA**  **n (%)^3^** |
| **Men** | 20-34 | 995,000 | 13 | 72,000 (7.24) | 2 (15.38) | 153,077 (15.38) |
| **Women** | 20-34 | 861,000 | 38 | 73,000 (8.48) | 3 (7.89) | 67,974 (7.89) |
| **Total** |  | **5,283,000** | **51** | **388,000** | **5** | **221,051** |
| **Stand.**  **Dif. %^4^**  **(95% CI)^5^** |  |  |  |  |  | **4.10**  **(4.04; 4.16)** |

^1^ Categorization of age groups was set by the data on employment of the German General population of the Federal Statistical Office Germany

^2^ BGP= Bavarian general population

^3^ Standardized proportion of the SEPIA study population

^4^ Standardized Difference (%)= Standardized Proportion of SEPIA (%) – Proportion of BGP (%)

^5^ 95% CI= 95% Confidence Interval

Table B3.2.2: Crude and age and sex-standardized comparison of the employment status of the SEPIA study population admitted to the GCPAR after 2000 not reliant on treatment and *the Bavarian General Population*

|  | | | | **Unemployment** | | |
| --- | --- | --- | --- | --- | --- | --- |
|  | Age groups^1^  [years] | **N BGP^2^** | **N Sepia** | **BGP^2^**  **n (%)** | **SEPIA**  **n (%)** | **Standardized**  **SEPIA**  **n (%)^3^** |
| **Men** | 20-34 | 995,000 | 16 | 72,000 (7.24) | 0 (0.00) | 0 (0.00) |
| **Women** | 20-34 | 861,000 | 13 | 73,000 (8.48) | 1 (7.69) | 66,230 (7.69) |
| **Total** |  | **5,283,000** | **29** | **388,000** | **1** | **66,230** |
| **Stand.**  **Dif. %^4^**  **(95% CI)^5^** |  |  |  |  |  | **-4.24**  **(-4.29; -4.20)** |

^1^ Categorization of age groups was set by the data on employment of the German General population of the Federal Statistical Office Germany

^2^ BGP= Bavarian general population

^3^ Standardized proportion of the SEPIA study population

^4^ Standardized Difference (%)= Standardized Proportion of SEPIA (%) – Proportion of GGP (%)

^5^ 95% CI= 95% Confidence Interval
